# RETRACTION: Uric Acid‐Induced Adipocyte Dysfunction Is Attenuated by HO‐1 Upregulation: Potential Role of Antioxidant Therapy to Target Obesity

**DOI:** 10.1155/sci/9753780

**Published:** 2026-03-27

**Authors:** Stem Cells International

RETRACTION: K. Sodhi, J. Hilgefort, G. Banks, C. Gilliam, S. Stevens, H. A. Ansinelli, M. Getty, N. G. Abraham, J. I. Shapiro, and Z. Khitan, “Uric Acid‐Induced Adipocyte Dysfunction Is Attenuated by HO‐1 Upregulation: Potential Role of Antioxidant Therapy to Target Obesity,” *Stem Cells International*, 2016, 8197325, https://doi.org/10.1155/2016/8197325.

The above article, published online on 22 November 2015 in Wiley Online Library (wileyonlinelibrary.com), has been retracted by agreement between the journal associate editor, Holm Zaehres; and John Wiley & Sons Ltd. The retraction has been agreed due to concerns identified in the Figures, both directly and on PubPeer [1]. On further investigation, the below issues have identified:

• The Control panels in Figures 1 and 3 appear identical
• The Uric acid, Uric acid + CoPP, and Uric acid + CoPP +SnMP panels in Figure 3b appear to be the identical to the Uric acid, UA + apocynin, and UA + CoPP panels in Figure 7b, though the panels in Figure 7 appear to have had the brightness adjusted. The indicated treatment for the panels in Figures 3 and 7 is different.
• The Actin bands in Figure 2a appear to be identical to the AKT bands shown in Figure 4c of [2]
• The Actin bands in Figure 2a appear to be identical to the Actin bands shown in Figures 1b and 2j of [2]
• The XO bands in Figure 2a appear to be identical to the HO‐1 bands shown in Figure 1b of [2]
• The Actin bands shown in Figure 6a appear identical to the AMPK bands shown in Figure 7c and the AKT bands shown in Figure 7b of [3]

Following an assessment of the concerns raised, together with the author’s response and associated raw data provided, the Editorial Board has concluded that the author’s explanation was insufficient and that the results and conclusions can no longer be considered reliable. The article is therefore retracted.

Zeid Khitan agrees with the retraction. The remaining authors were unresponsive.
